# Frailty in Patients With Chronic Kidney Disease Stage Five

**DOI:** 10.7759/cureus.43787

**Published:** 2023-08-20

**Authors:** Jerry Joseph, Murugesan Vellaisamy, Thirumavalavan Subramanian, Edwin Fernando, Thirumalvalavan Kaliaperumal, Srinivasaprasad ND, Sujith Surendran, Poongodi Annadurai, Nived Haridas

**Affiliations:** 1 Nephrology, Government Stanley Medical College and Hospital, Chennai, IND

**Keywords:** ckd5, renal replacement therapy, comorbidities, hemodialysis, chronic kidney disease (ckd), frailty

## Abstract

Aim

To study the prevalence of frailty in patients with chronic kidney disease stage 5 (CKD5) and to assess coexisting factors associated with frailty in chronic kidney disease.

Patients and methods

We studied the prevalence of frailty in CKD5 patients from November 2021 to November 2022. CKD5 patients over 18 years of age were included. Patients on maintenance hemodialysis and CKD5 patients on pre-dialysis care were included. Patients with active infection and significant morbidity were excluded. We performed a history and clinical examination and recorded laboratory data.

We performed frailty assessments using modified Fried’s criteria. Frailty was defined based on previously validated Fried’s criteria, which included 1. Slowness, 2. Weakness, 3. Unintentional weight loss, 4. Exhaustion, 5. Low physical activity. A patient is considered frail if three or more components are present. We evaluated the prevalence of frailty in pre-dialysis and dialysis care participants and the association of frailty with coexisting factors.

Results

Of the 139 patients, 84 were on thrice-weekly hemodialysis, and 55 were on pre-dialysis care. We found the prevalence of frailty to be 41%. The prevalence of frailty was similar in patients on pre-dialysis care and hemodialysis. The prevalence of frailty in hemodialysis patients and those in pre-dialysis care was 43% and 40%, respectively. The prevalence of frailty among the elderly (over 55) was 82%. The prevalence of frailty among diabetes patients was 75%. Factors with a statistically significant association with frailty included old age (*p* < 0.005), native kidney disease (*p* < 0.005), edema (*p* < 0.001), intradialytic hypotension (*p* = 0.002), and various comorbidities like diabetes (*p* < 0.001), heart failure (*p* < 0.001), coronary artery disease (*p *= 0.001), and cerebrovascular accidents (*p* = 0.016). We observed no significant association with the duration of chronic kidney disease (CKD) (*p* = 0.458), duration of dialysis (*p* = 0.838), or body mass index (BMI) (*p *= 0.267). The most commonly reported frailty components were exhaustion (61.9%), low physical activity (61.2%), and weak handgrip (55.4%).

Conclusion

Frailty is a marker of increased vulnerability to adverse outcomes. A significant proportion, 41% of CKD5 patients, are frail. Dialysis does not affect the prevalence of frailty in CKD5 patients. Old age, native kidney disease, edema, intradialytic hypotension, and comorbidities like diabetes, heart failure, coronary artery disease, and cerebrovascular accident are significantly associated with frailty in CKD5 patients. CKD patients with those conditions should receive special care to reduce the development of frailty.

## Introduction

Frailty is a construct that gerontologists initially designed to describe cumulative decline across multiple physiological systems that occur with aging [1]. The frailty phenotype incorporates disturbances across interrelated domains to identify individuals with diminished functional reserve, placing them at risk of adverse health outcomes [1-3]. Across diverse populations, frailty is associated with future risk of disability, hospitalization, and premature death [1,4-6]. Aging is closely related to the prevalence of frailty [1]. But, even for the elderly, exercise and strength training have reduced physical frailty [1,7].

Several studies have reported a greater prevalence of frailty among those with CKD, especially end-stage renal disease (ESRD), than those without CKD. This higher prevalence of frailty is due to disease-related and disease-associated conditions such as protein energy wasting (PEW), anemia, inflammation, acidosis, and hormonal disturbances [8]. There is an overlap in the pathophysiology and symptomatology of CKD and frailty. This overlap increases the probability of a massive burden of frailty in the CKD population.

The prevalence of frailty among the general population is around 2.8%, but among persons with moderate to severe CKD, the prevalence can be as high as 20.9% [9]. In the literature, the prevalence of frailty has been reported to be as high as 50%-80% among patients with ESRD [10,11]. The prevalence of frailty among CKD patients over and under the age of 65 years has been reported to be 43.6% and 27.5%, respectively. In recent times frailty has emerged as a good predictor of prognosis in populations with ESRD. It has been reported that frail patients on hemodialysis have 2.6 times a mortality risk and 1.4 times a risk of hospitalization independent of age, gender, comorbidity, and disability compared to frail patients [12].

Our study seeks to estimate the prevalence of frailty in patients with CKD5 and to assess associated factors. As there are a limited number of studies evaluating frailty in patients with CKD5, our study can be instrumental in sensitizing the nephrology community toward identifying the prevalence of frailty in patients with CKD5, which may translate into better management and improved clinical outcomes.

## Materials and methods

Study design

This is a cross-sectional observational study involving outpatients and inpatients from dialysis units of a tertiary referral center. The study was approved by the Institutional Ethics Committee at Government Stanley Medical College and Hospital, Chennai, with the approval number ECR/131/Inst/TN/2013/RR-22. 

Patient selection and data collection

We recruited all cases of CKD5 among those over age 18, including those on maintenance dialysis (CKD5D) and those not on maintenance dialysis (pre-dialysis care). We defined the presence of CKD5 as an estimated glomerular filtration rate (GFR) by serum creatinine based on an MDRD equation (eGFRcr) of <15mL/min/1.73m2. We defined maintenance dialysis as a minimum of thrice weekly hemodialysis for the previous three months. Patients with active infection, those with significant morbidity and disability, and those without a reliable bystander were excluded from the study. Data on demographic characteristics, clinical features, comorbidities, and biochemical parameters were recorded.

**Figure 1:**
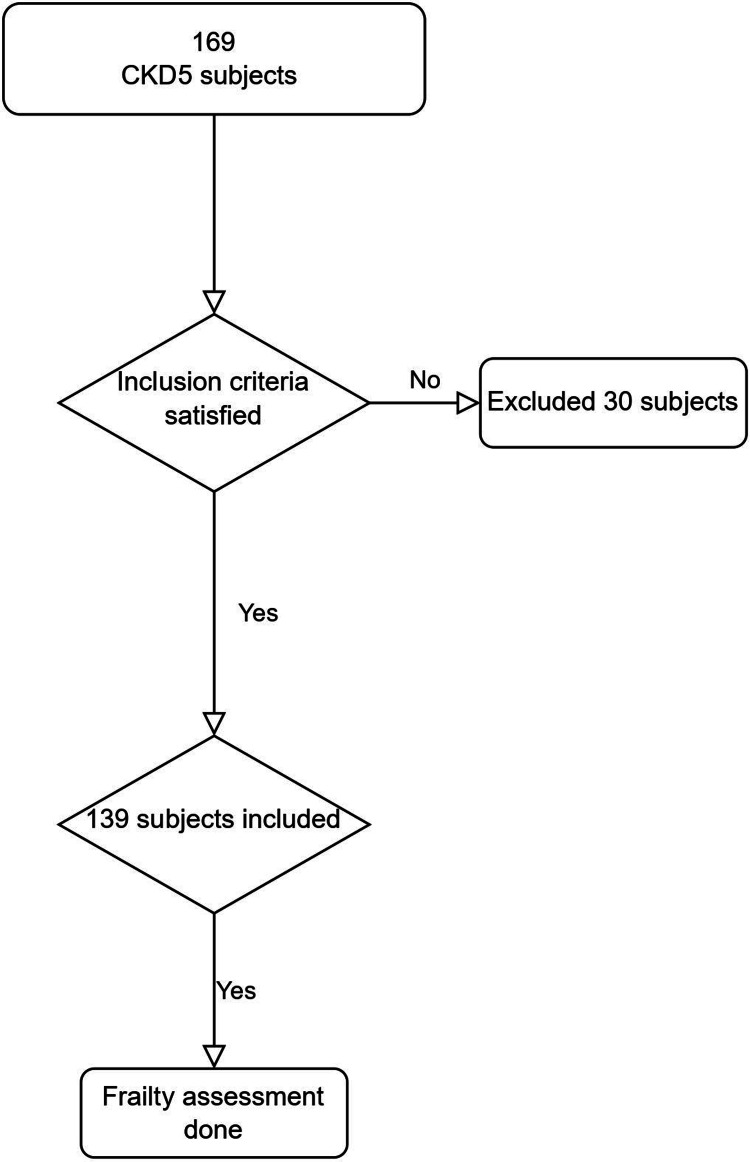
Study flow diagram

From November 2021 to November 2022, we assessed 169 CKD 5 patients. We excluded 25 patients who did not complete the frailty assessment and five ineligible, leaving 139 participants for analysis (Figure 1).

Assessment of frailty

**Figure 2:**
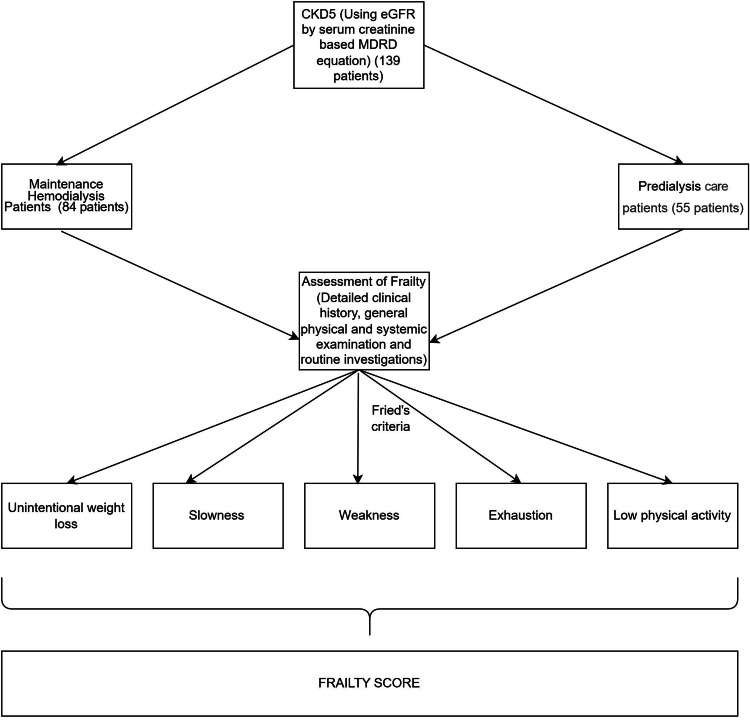
Methodology of the study

We defined frailty using a slight modification of criteria initially established by Fried et al. [2] for physical activity and self-reported weight loss, which were based on data from the Cardiovascular Health Study (CHS), a community-based study of adults aged 65 years and older (Figure 2). According to our definition, at least three of the following five conditions must be present for someone to be considered frail: weakness, unintentional weight loss, slow gait, exhaustion, and low activity (Table 1). We defined an intermediate frailty phenotype as having one or two of these conditions. We defined weight loss as an unintentional, self-reported, 5 kg weight loss during the previous six months [1]. We measured grip strength in each participant’s dominant hand using an analog hand dynamometer (GBEX Co. Ltd., Republic of Korea) and analyzed the maximal reading from three consecutive efforts. We defined weakness as a grip strength less than the lowest sex and body mass index (BMI)-specific 20th percentile score in the CHS [2,13]. We performed usual gait-speed assessments twice per participant when walking at his or her average pace over a 15-feet course, recording the best of the two times. Slowness was based on the time to walk 15 feet, adjusted to gender and height. We used the mean cut-off that Fried et al. gave [2]. 

**Table 1:** Operational definitions (a) Exhaustion items on CES-D are "I felt everything I did was an effort" and "I could not get going." CES-D: Centre for Epidemiological Studies Depression

	Definition
Weight loss	Self-reported unintentional weight loss of 5kg in previous six months
Weakness	Lowest sex and BMI-specific 20^th^ percentile grip strength
Low activity	Self-reported exercise of less than once a week
Exhaustion	Positive response to either exhaustion item on CES-D (a)
Slowness	Low sex and height specific 20^th^ percentile walking pace assessed over a 15-foot course

Exhaustion is defined as a positive response to either exhaustion item on the Centre for Epidemiological Studies Depression (CES-D) scale in CHS [12]. We assessed physical activity from self-reported exercise habits similar to a prior study of frailty in participants with ESRD [1,10]. Participants were considered to have low physical activity if they reported never exercising or exercising less than once weekly. CHS defined physical inactivity as the lowest sex-specific quintile kilocalories per week.

Data management and statistical analysis

We entered the data into a computer-based spreadsheet and analyzed it using SPSS version 20.0 (IBM Corp., Armonk, NY). The statistical analysis consisted of calculating means and proportions. We used appropriate statistical tests of significance-like, like the Chi-square test (Fisher’s exact test wherever applicable) and Student’s t-test-to test the association of frailty with various patient characteristics.

## Results

Characteristics of the cohort

The mean age of the cohort was 45; 72% of participants were men, 28% were women; 31.6% had diabetes; 85% had hypertension; and 21% had a history of heart failure. The mean duration of CKD was 26 months, and 60.4% of participants were on renal replacement therapy (hemodialysis). The mean duration of renal replacement therapy was seven months (Table 2).

**Table 2:** Patient characteristics

		All	Frail		P-value
		All (N =139)	No (n =81,58.3%)	Yes (n = 58, 41.7%)	
Age (in years)	Mean (S.D)	44.9+11.9	39.59 + 10.434	52.26 + 9.747	<0.005
Gender	Male	100 (72)	64 (79)	36 (62)	0.028
	Female	39 (28)	17 (21)	22 (38)	
Education	<10^th^ grade	72 (52)	37 (46)	35 (60)	0.088
	>=10^th^ grade	67 (48)	44 (54)	23 (40)	
Physical examination					
BMI (kg/m^2)	Mean (S.D)	20.89 + 2.71	21.111 + 3.19	20.593 + 1.8	0.267
SBP (mm Hg)	Mean (S.D)	144.54 + 15.69	145.58 + 14.387	143.1+ 17.38	0.361
Pallor		34 (24)	23 (28)	11 (19)	0.202
Edema		14 (10)	0	14 (24)	<0.001
Native kidney disease					<0.005
Diabetic Nephropathy		44 (31.7)	11 (14)	33 (57)	
IgA Nephropathy		9 (6.5)	9 (11)	0	
Others		86 (61.8)	61 (75)	25 (43)	
Duration of illness					
Duration of CKD (in months)	Mean (S.D)	25.76 + 28.72	24.23 + 21.001	27.91 + 37.021	0.458
Duration of dialysis (in months)	Mean (S.D)	7.07 + 7.69	7.19 + 8.038	6.91 + 7.253	0.838
Laboratory values					
Hemoglobin (g/dL)	Mean (S.D)	9.84 + 1.36	10.217 + 1.25	9.517 + 1.44	0.062
Urea (mg/dL)	Mean (S.D)	116.11 + 32.3	114.19 + 31.01	118.81 + 34.11	0.407
Creatinine (mg/dL)	Mean (S.D)	8.21 + 2.07	8.42 + 1.82	7.936 + 2.37	0.177
Albumin (g/dL)	Mean (S.D)	3.47 + 0.37	3.528 + 0.35	3.405 + 0.40	0.057
Comorbidity	Diabetes mellitus	44 (32)	11 (14)	33 (57)	<0.001
	Hypertension	118 (85)	66 (81)	52 (90)	0.185
	Heart failure	29 (21)	0	29 (50)	<0.001
	Coronary artery disease	7 (5)	0	7 (12)	0.001
	Cerebrovascular accident	4 (3)	0	4 (7)	0.016
	Thyroid disorder	15 (11)	6 (7)	9 (16)	0.129
Renal Replacement	Yes	84 (60)	48 (59)	36 (62)	0.738
	No	55 (40)	33 (41)	22 (38)	
Intradialytic hypotension					0.002
	No	59 (42.4)	41 (50.6)	18 (31)	
	Yes	25 (18)	7 (8.6)	18 (31)	

Prevalence of frailty

**Figure 3:**
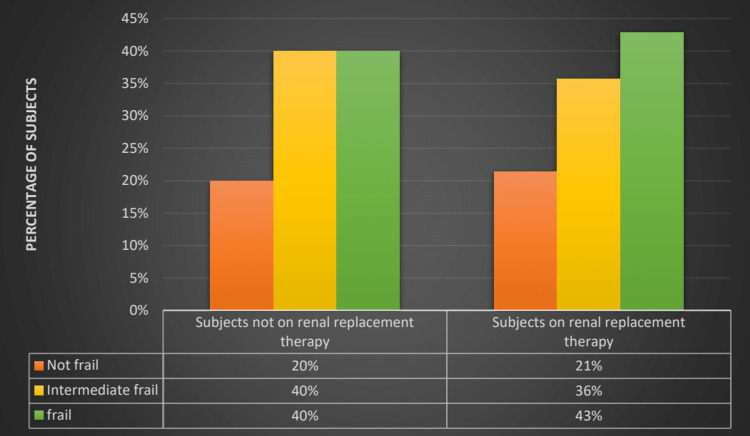
Prevalence of frailty and intermediate frailty in patients on renal replacement therapy and pre-dialysis care

Fifty-eight participants met the criteria for frailty, resulting in a prevalence of 41%. The prevalence of intermediate frailty was 37%, and 21% were not frail. For the male and female participants, the prevalence of frailty was 36% and 56%, respectively. The prevalence of frailty among patients over and under 55 years was 82% and 29.2%, respectively. The prevalence of frailty among hemodialysis patients and those in pre-dialysis care was 43% and 40%, respectively (Figure 3). For diabetic patients, the prevalence of frailty was 75% compared to 26% for non-diabetic patients. The most common frailty components in the cohort were exhaustion (61.9%), low physical activity (61.2%), and weak handgrip (55.4%).

Characteristics of frail individuals

Frail patients were more likely to be elderly. The mean age of frail participants was 52.6 years, while that of non-frail participants was 39.6. The prevalence of frailty among patients over and under 55 was 82% and 29.2%, respectively. Frailty was more common in females than males (56% vs. 36%). Participants beyond tenth grade were less frail than those with less education (34.4% vs. 48.6%). Frail participants were more likely to have diabetes, heart failure, coronary artery disease, and cerebrovascular accidents. Native kidney disease among frail patients was different from those who were not frail. Diabetic nephropathy was the major cause of kidney disease among frail patients. The cause of native kidney disease was unknown for most non-frail patients. Participants with edema were found to be frail. Frail individuals had lower hemoglobin and albumin. The prevalence of frailty in patients on hemodialysis and those on pre-dialysis care was similar. But participants with frequent intradialytic hypotension episodes were found to be frailer. 

Factors associated with frailty

Older participants were frailer than younger participants (p < 0.005). Participants with comorbidities were trailer than those without. The prevalence of frailty was greater in patients with diabetes (p < 0.001), heart failure (p < 0.001), coronary artery disease (p = 0.001), and cerebrovascular accidents (p = 0.016). The prevalence of frailty increased as comorbidities increased (p < 0.001). Frailty was greater in those with significant edema (p < 0.001) and those with intradialytic hypotension (p = 0.002). Most frail patients had diabetic nephropathy, while in the majority of non-frail patients, native kidney disease is not known (p < 0.005).

Though we found female gender and low education to have a higher association with frailty, that association was not statistically significant (p = 0.028). Frail patients had less BMI than those who were not frail, but this was not statistically significant (p = 0.267).

Frailty was not associated with the duration of chronic kidney disease (p = 0.458) or the duration of hemodialysis (p = 0.838). Prevalence of frailty was similar in CKD5 patients irrespective of whether they were on renal replacement therapy (p = 0.738). Frail patients were more anemic (p = 0.062) and hypoalbuminemic but not to a statistically significant extent (p = 0.057).

## Discussion

Frailty, the state of increased vulnerability to physical stressors due to the progressive degeneration of multiple physiological systems, is common in those with CKD [14]. The prevalence of frailty in older adult populations is 11%. Some studies report that frailty prevalence is greater than 60% in dialysis-dependent CKD populations [9-11,14]. Various associated factors like advanced age, inflammation, anemia, hypoalbuminemia, and comorbidities like diabetes, hypertension, and heart failure have been proposed as the causes for the increased prevalence of frailty in the CKD population.

In our study of CKD5 patients, the prevalence of frailty was 41%. This prevalence is six times greater than the reference population used to establish the frailty phenotype, which was, on average, 20 years older [1,2]. The most common frailty components in individuals with CKD5 were exhaustion, low physical activity, and weak handgrip. Among the factors associated with frailty were age, comorbidities (diabetes, coronary artery disease, cerebrovascular accidents), number of comorbidities, nature of native kidney disease, significant edema, and intradialytic hypotension.

Forty-one percent of our CKD5 patients were frail. The prevalence of frailty was not different for those on maintenance hemodialysis and those on conservative treatment. The prevalence of frailty in patients on hemodialysis and pre-dialysis patients was 43% and 40%, respectively. The prevalence of frailty was higher in CKD patients than in the general population. Various studies show the prevalence of frailty in CKD to be between 15% to more than 80% [9-11]. It has also been shown that the prevalence of frailty increases as the stage of CKD advances. Previous studies showed that the prevalence of CKD in patients on maintenance hemodialysis was as high as 80% [10,11,13]. But in our study prevalence of frailty in patients on maintenance hemodialysis was only 42%. This may be because our patients were on hemodialysis for less time than those in other studies. The mean duration of hemodialysis for our patients was seven months compared to 2.5 years in a study conducted by Yadla et al. [15]. The prevalence of frailty in our CKD patients not on dialysis was higher than in other studies [16]. This may be because other studies mainly included participants in all stages of CKD, whereas our study included participants with CKD5 only. 

Elderly participants were frailer than younger ones. Frailty is associated with sarcopenia and dynapenia [17]. Older people have reduced muscle mass and strength due to morbidity, malnutrition, or physical inactivity. Skeletal muscle mass is among the most critical predictors of muscle strength or physical performance. With advancing age, the rate of decrease in muscle strength is more than the rate of loss of muscle mass. The power of muscles can decline even though muscle mass is maintained or increased. Physical performance is defined as the capability to conduct normal daily physical activities. Reduced physical performance leads to frailty. Physical performance and mortality are associated more with muscle strength than muscle mass. In the elderly, all these factors may lead to frailty [17].

Our study shows that women were more likely to become frail. Female gender confers an intrinsic risk of frailty as females have lower lean mass and strength than age-matched men; therefore, losing lean body mass with aging is more likely to cross the threshold necessary for frailty [12,18].

In our study, participants with lesser education were frailer. Most of our patients were from lower socioeconomic status and were unemployed or doing unskilled labor. So, we couldn’t assess the relationship between quality of life with frailty in CKD. Lee et al. reported that patients with lesser education and those unemployed were frailer [19]. Nixon et al. said that frailty is independently associated with high symptom burden and poor health-related quality of life (HRQOL) in CKD [20].

In our study, frailty was significantly associated with comorbidities like diabetes, coronary artery disease, cerebrovascular accident, and heart failure. These patients have an underlying inflammatory state. They also have reduced physical activity. When a CKD patient in accelerated metabolic aging associated with protein-energy wasting, anemia, acidosis, chronic inflammation, oxidative stress, and vascular calcification has these comorbidities, they have an increased chance of developing frailty [16,19]. In their study, Lee et al. noticed that participants with cardiac and cerebrovascular disease were frailer [19].

We also found intradialytic hypotension to be associated with frailty. Intradialytic hypotension is associated with poor long-term outcomes. Patients with intradialytic hypotension show increased mortality [21] and an increased rate of cardiac-wall motion abnormalities during dialysis, the so-called myocardial stunning [22].

Our study has several limitations. First, the cross-sectional design makes it difficult to conclude the temporal relationship between CKD and frailty. Second, the generalizability of our findings may be limited as the study was conducted in only one center with a small population. Third, our study has a high proportion of male patients with CKD. Further studies are needed to confirm these findings in women with CKD. Fourth, the self-reported items in the frailty assessment may be responsible for recollection/reporting bias, leading to inaccurate estimates. Fifth, even though we have tried to find the association of most factors with frailty, we may have missed some chronic conditions like underlying malignancies, autoimmune conditions, and chronic gastric problems like ulcers and reflux diseases. Last, we haven’t assessed the relation of factors like drug compliance and diet that may be associated with frailty in CKD.

## Conclusions

The presence of CKD makes the clinical syndrome of frailty even more vulnerable to negative consequences, and it shows a reciprocal interaction with uremia, multisystem dysregulation, inflammation, and infection. A high occurrence of frailty in CKD5 patients is mainly associated with old age, comorbidities, edema, and intradialytic hypotension. Frailty will play a significant role in clinical care as dialysis and pre-dialysis CKD patients increase. Frailty is associated with poor clinical outcomes, quality of life, and disability, and it plays an integral part in managing CKD patients. Thus, prompt identification of frailty can affect effective management and improve clinical outcomes in CKD patients.
